# Comparable Risk of Suicidal Ideation between Workers at Precarious Employment and Unemployment: Data from the Korean Welfare Panel Study, 2012–2017

**DOI:** 10.3390/ijerph16162811

**Published:** 2019-08-07

**Authors:** Woorim Kim, Myung Ki, Minjae Choi, Areum Song

**Affiliations:** Department of Preventive Medicine, College of Medicine, Korea University, Seoul 02841, Korea

**Keywords:** employment transition, employment status, precarious employment, suicide ideation

## Abstract

Precarious employment and unemployment are important factors that impact suicidal behavior. This study investigated (1) how employment transitions among permanent employment, precarious employment, and unemployment are associated with suicidal ideation in working employees and compared (2) whether individuals transitioning among these three states were more vulnerable than those remaining. Using nationally representative longitudinal data between 2012–2017, a total of 25,862 adults aged 25 to 59 years old without a record of suicidal ideation were included at baseline. Transitions in employment status (permanent work, precarious work, or unemployment) and suicidal ideation were analyzed using hierarchical logistic regression models. Compared to the “permanent to permanent” group, individuals in the “permanent to precarious” (odds ratio (OR) 1.74, 95% confidence interval (95% CI) 1.29–2.35], “permanent to unemployment” (OR 1.97, 95% CI 1.32–2.96), “precarious to precarious” (OR 1.86, 95% CI 1.21–2.85), and the “precarious to unemployment” (OR 1.43, 95% CI 1.05–1.95) groups had higher odds of suicidal ideation. The magnitude of such odds was generally higher than that of individuals at annual unemployment or precarious states. The results show that adults moving in and out of different employment states have higher odds of suicidal ideation than individuals at annually static precarious or unemployment status.

## 1. Introduction

Suicide is a notable public health issue associated with human, social, and economic losses, estimated to have caused approximately 788,000 deaths annually by the World Health Organization (WHO) [1,2]. Suicide is particularly important in South Korea, located in East Asia, as it ranks second among Organization for Economic Cooperation and Development (OECD) countries for suicide rates, at 25.8 deaths per 100,000 population [3]. In fact, suicide is the fifth leading cause of death overall and the first leading cause of death in ages 10 to 39 years old [4]. Many factors, including demographic factors, psychiatric disorders, physical illness, socioeconomic status, alcohol or drug abuse, and family histories have been noted to impact suicide and suicidal behavior [5].

Of these factors, the effect of unstable employment, including unemployment and precarious employment, requires specific attention as the number of workers in unstable, temporary jobs have increased globally [6]. Employment status is an important component of socioeconomic status, which reflects the amount of resource availability, social network, status, and psychosocial process related to the stress of an individual [7]. Considering that unemployment can often lead to financial strains and relative social exclusiveness through interruption of personal networks while precarious employment is generally characterized by low wages, few promotion opportunities, and relative unstableness, it is important to understand the potential impact of unstable employment on suicidal behavior in economically active individuals [8,9].

Previous studies have reported an association between unemployment and suicidal behavior, with the risk of suicide being highest in the first years of unemployment [10,11]. For instance, one study revealed that the unemployed report high risks, with the degree of risk being high in early periods [11]. In contrast, some studies reported that unemployment produced a more pronounced adverse effect on long-term than short-term unemployment, suggesting the possibility of adversity accumulation [12,13]. Similarly, a cohort study in Canada also reported that part-time workers and the unemployed are more likely to attempt suicide than full-time workers [14]. With regard to precarious work, numerous studies found that precarious work is strongly related to adverse mental health, including psychological distress, depression, and suicide [1,15,16]. Specifically, studies revealed that precarious employment is associated with increased suicidal ideation [17,18]. Likewise, a study conducted in Germany reported high likelihoods of suicide deaths in temporary workers [19].

Collectively, these studies found an adverse effect of unemployment or precarious employment on suicide. However, many were cross-sectional in design and often provided labor market classification based on an individual’s status at one-time point instead of taking into consideration transitions in employment [1]. Such studies incorporating employment status without a referring origin may be limited in view as the economic activity of an individual is bound to change over one’s life course [20]. Moreover, many of these studies also did not take into consideration unemployment and precarious employment concurrently, neglecting heterogeneity among the employed. Therefore, the current study aimed to fill this research gap by examining (1) how employment transitions between permanent employment, precarious employment, and unemployment affect suicidal ideation in working employees and to compare (2) whether individuals transitioning between these three states are more vulnerable than individuals remaining at static states.

## 2. Materials and Methods

### 2.1. Study Population and Data

This study applied the longitudinal case study design, using data from the Korea Welfare Panel Study (KOWEPS), 2012 to 2017. The KOWEPS is a panel study of a nationally representative sample of South Korean households conducted by the Korea Institute for Health and Social Affairs (KIHASA) [21]. Trained interviewers conducted face-to-face interviews annually to households selected using a stratified multistage probability sampling design using structured questionnaires covering an array of subjects, including employment status, health care use, demographic and socioeconomic characteristics, subjective health status, and social service needs. All individuals aged 15 years or above were included in the KOWEPS data and repeatedly measured at each wave of data collection.

**Figure 1:**
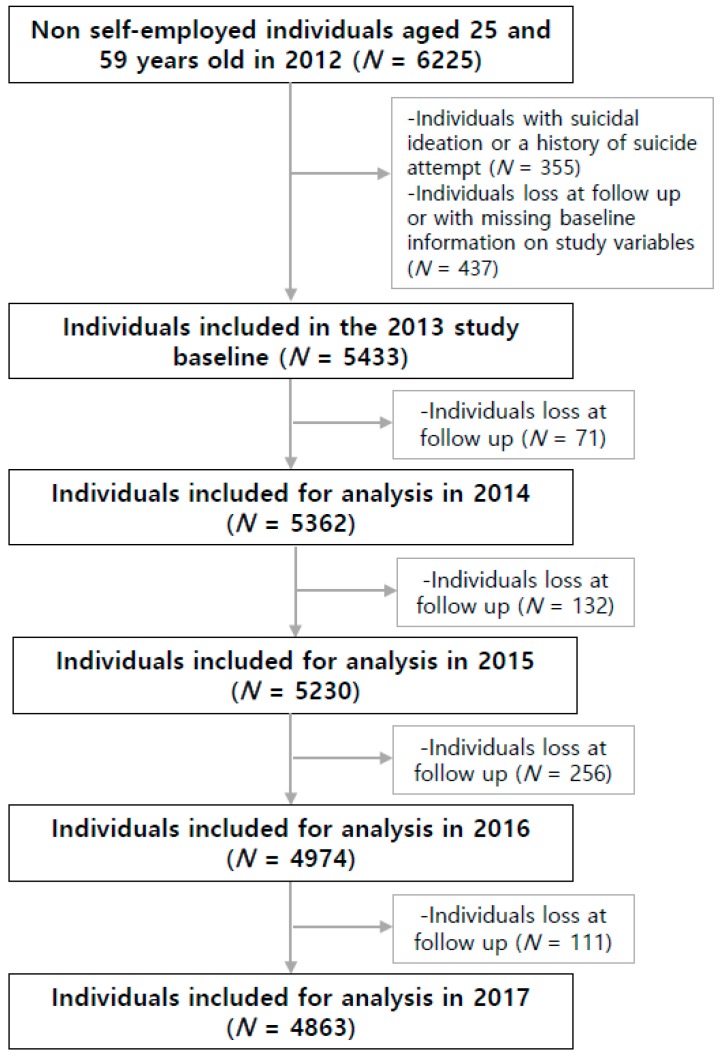
Study profile.

This study used the 2012 to 2017 data as these waves of data included information on suicidal ideation. In the 2012 baseline data, all individuals with a record of suicidal ideation or attempt were excluded and followed up to the 2013 data. Afterwards, all individuals aged 25 years old or above recorded in the 2013 data were included in the analysis to compose the final study population of 25,862 cases (Figure 1).

### 2.2. Outcome Variable

The outcome variable of this study was suicidal ideation. The KOWEPS data investigates suicidal ideation based on the question “Did you seriously think about committing suicide in the past one year?” In this study, all study participants who answered “yes” to the question above were categorized as reporting suicidal ideation and vice versa.

### 2.3. Main Predictor

The main predictor variable of this study was employment status change between 2012 and 2017. Changes in employment were categorized into the following nine groups: “permanent to permanent”, “permanent to precarious”, “permanent to unemployment”, “precarious to permanent”, “precarious to precarious’”, “precarious to unemployment”, “unemployment to permanent”, “unemployment to precarious”, and “unemployment to unemployment”. The permanent group encompassed all waged, full-time workers hired directly by their employers. The non-permanent group included precarious workers under fixed-term contracts and part-time workers. In Korea, fixed-term contracts can last up to a maximum of 2 years. Self-employed individuals, referring to individuals with their own business, were excluded from the analysis. 

### 2.4. Covariates

Demographic, socioeconomic, and health related variables were incorporated as covariates in this study. Specifically, the covariates included sex (male and female), age (25–34, 35–44, and 45–59), region (metropolitan cities, urban, or rural), education level (high school or below and university or above), marital status (single, separated, and married), income (low, medium, and high), disability status (no and yes), chronic disease status (no and yes), depression (no and yes), and year (2013, 2014, 2015, 2016, and 2017). Regarding income, equalized household income was used because it is commonly used for measuring household income in Korea while also being recommended by the OECD [22]. Utilizing equalized household income allows income comparability among households of different sizes as it is calculated by dividing household income by the square root of the number of household members.

### 2.5. Statistical Analyses

The general characteristics of the study population were examined using Pearson’s chi-square tests. In the current study, employment transition was conceptualized as occurring over 2 years, at the time point of the first measurement each year and the year afterwards. As the KOWEPS data are hierarchically organized and longitudinal in nature, the association between changes in employment and suicidal ideation was investigated using hierarchical logistic regression models fitted using the GLIMMIX procedure. This analysis is adapted for hierarchical modelling of multiple exposures with a dichotomous or polytomous outcome, in which multilevel models can account for the correlated structure of hierarchical data while also controlling for the non-independence of observations within groups when investigating individual level outcomes [23]. Additionally, this study compared the results of the analysis on employment transition and suicidal ideation with the results of the analysis on static employment type and suicidal ideation. Through comparison, this study aimed to identify specific patterns in employment transition that may increase the likelihood of suicidal ideation. All analyses were conducted using the SAS software, version 9.4 (SAS Institute, Cary, NC, USA).

## 3. Results

A total of 639 (2.5%) cases showed suicidal ideation. The general characteristics of the study population are shown in Table 1. Of a total of 25,862 cases analyzed, the highest number of observations were found in the “permanent to permanent” group, followed by the “unemployment to unemployment” group, whereas the fewest observations were found in the “unemployment to permanent” group.

Table 2 presents the results of the analysis investigating the association between changes in employment status (a total of nine possible transitions with three transitions per origin) and suicidal ideation. Compared to the “permanent to permanent” group, individuals in the “permanent to precarious” (odds ratio (OR) 1.74, 95% confidence interval (95% CI) 1.29–2.35), “permanent to unemployment” (OR 1.97, 95% CI 1.32–2.96), “precarious to precarious” (OR 1.86, 95% CI 1.21–2.85), and the “precarious to unemployment” (OR 1.43, 95% CI 1.05–1.95) groups showed higher odds of suicidal ideation. The magnitude of such odds was generally higher than that of individuals at annual unemployment (OR 1.36, 95% CI 1.05–1.78) or precarious (OR 1.52, 95% CI 1.17–1.99) status.

## 4. Discussion

This study demonstrated that precarious workers and the unemployed report higher likelihoods of suicidal ideation, while adjusting for known risk factors including gender, chronic disease, and pre-existing depression. The results are in line with previous studies as unemployment has been consistently associated with increased mortality from all-causes and suicide [24]. A 3 year follow-up study on a representative sample of the US population suggested that unemployed men and women report high suicidal risks than economically active individuals [25]. A meta-analysis on 16 studies similarly concluded that unemployment was associated with elevated risks of suicide [10]. Moreover, unemployment has been identified to induce self-destructive behaviors and increase suicide risk [26]. In fact, a study on Korean workers also revealed that individuals moving from permanent to unemployment status expressed higher odds of depression [6]. As for precarious employment, a longitudinal cohort study of Korean women concluded that transitions into precarious employment can escalate the likelihood of depressive symptoms [27]. Another longitudinal study also reported similar results for suicidal ideation, along with several other cross-sectional studies presenting identical trends [1,17,18].

The relationship between unemployment and increased risk of suicidal ideation may be partially explained by the tendency of unemployment to induce financial distress while reducing social networks, as jobs often serve to provide financial and social resources [28]. In fact, job displacement in many cases will inevitably result in a reduction of earnings and available economic resources [29]. Furthermore, unemployed individuals are likely to face cessations in social networks and possibly a loss of social roles, which, in turn, can lead to decreased levels of self-regard and social standing [30,31].

This study demonstrated that employment transitions from permanent to non-permanent employment status can elevate odds of suicidal thought, which is in line with previous studies conducted in Korea, Japan, and Western and Nordic countries [1,14,15,17,18,32]. This finding suggests that accumulative changes in employment status may act to induce comparatively higher levels of suicidal ideation in individuals. A review found that mortality risk was highest among the unemployed at 5 year follow up, while it decreased at the 10 year follow-up session [33]. Likewise, individuals may report a higher risk of mental disorders between 3 months and 1 year compared to an immediate period following unemployment due to the increasing sense of hopelessness [34,35]. Adding to the currently available evidence on this subject, the finding of this study notably demonstrates that workers swinging between employment states may be more vulnerable to suicidal ideation than individuals at annual precarious or unemployment status. This finding supports the results of previous studies, which found that precarious employment may be more harmful than the actual experience of unemployment over consecutive years [36]. The results are noteworthy because Korea ranks as the second highest country among OECD countries in suicide rate and the amount of precarious work has continuously increased to comprise approximately 50% percent of the entire labor force [3,37].

Precarious work has also been associated with suicidal ideation to a similar degree with unemployment. This result suggests that categorizing individuals into only the employed and unemployed group may not be adequate as permanent and precarious employees exhibit different levels of suicidal ideation. Characteristics of precarious employment, such as hazardous working conditions and insecurity, may work to negatively affect the mental health of workers. With employment status being a crucial component of socioeconomic status, precarious work is often characterized by high levels of job insecurity, low control or powerlessness, and poor working conditions [7]. Specifically, precarious workers are reported to generally receive lower wages and gain fewer promotion opportunities than their employed counterparts [38]. In fact, distribution of wages and household income have been reported to be different between precarious and permanent workers, suggesting socioeconomic inequalities [39]. Moreover, as labor supervisory regimes are often less strictly applied to precarious than permanent workers, few precarious workers are able to work under the maintenance of minimum labor standards [39]. This infers that precarious workers are less likely to receive regular health checks and support from occupational and environmental health physicians. Unsurprisingly, precarious workers are likely to work with less social security protection, are often uncovered by unemployment insurance, and often not paid for overtime work [18]. Such characteristics of uncertain working conditions may increase emotional distress, which are identified risk factors for self-destructive behaviors [19].

This study is not without its limitations. First, the issue of reverse causality cannot be completely ruled out because, even though this study utilized longitudinal data, this study measured changes in employment, allowing the possibility of a partial overlap in the period of exposure and outcome. Second, recall bias for household income may have resulted from self-reports. Third, this study could only measure changes in employment status over two consecutive years. Although the presented results can show better inferences concerning behavioral changes across time than cross-sectional analyses, the findings are a reflection of short-term changes. Further studies incorporating a longer time frame would be beneficial. However, despite the limitations stated above, this study was unique as it included not only permanent and precarious employees but also the unemployed.

## 5. Conclusions

Individuals transitioning from permanent to precarious or unemployment status and from precarious to unemployment status showed higher odds of suicidal ideation than individuals constantly in permanent employment. These individuals also generally showed higher likelihoods of suicidal ideation than individuals at annually static precarious or unemployment status. Thus, adequate legal protection and social safety nets are necessary to focus on unstable employment as it can lead to mental problems.

## Figures and Tables

**Table 1:** General characteristics of the study population.

	*n*	Suicide Ideation *n* (%)				*p*-Value
		No		Yes		
**Employment transition**						
Permanent → permanent	9506	9418	(99.1)	88	(0.9)	<0.0001
Permanent → precarious	594	580	(97.6)	14	(2.4)	
Permanent → unemployment	452	445	(98.5)	7	(1.6)	
Precarious → permanent	834	815	(97.7)	19	(2.3)	
Precarious → precarious	5166	5018	(97.1)	148	(2.9)	
Precarious → unemployment	953	911	(95.6)	42	(4.4)	
Unemployment → permanent	427	419	(98.1)	8	(1.9)	
Unemployment → precarious	1161	1114	(96.0)	47	(4.1)	
Unemployment → unemployment	6769	6503	(96.1)	266	(3.9)	
**Employment status**						
Permanent	10,767	10,652	(98.9)	115	(1.1)	<0.0001
Precarious	6921	6712	(97.0)	209	(3.0)	
Unemployed	8174	7859	(96.2)	315	(3.9)	
**Sex**						
Male	11,507	11,277	(98.0)	230	(2.0)	<0.0001
Female	14,355	13,946	(97.2)	409	(2.9)	
**Age**						
25–34	5241	5157	(98.4)	84	(1.6)	<0.0001
35–44	8847	8685	(98.2)	162	(1.8)	
45–59	11,774	11,381	(96.7)	393	(3.3)	
**Region**						
Metropolitan cities	12,259	11,934	(97.4)	325	(2.7)	0.0285
Urban	10,523	10,265	(97.6)	258	(2.5)	
Rural	3080	3024	(98.2)	56	(1.8)	
**Education level**						
High school or below	13,240	12,774	(96.5)	466	(3.5)	<0.0001
University or above	12,622	12,449	(98.6)	173	(1.4)	
**Marital status**						
Single	5090	4950	(97.3)	140	(2.8)	<0.0001
Separated *	2383	2202	(92.4)	181	(7.6)	
Married	18,389	18,071	(98.3)	318	(1.7)	
**Income**						
Low	8574	8148	(95.0)	426	(5.0)	<0.0001
Medium	8623	8492	(98.5)	131	(1.5)	
High	8665	8583	(99.1)	82	(1.0)	
**Disability**						
No	24,497	23,950	(97.8)	547	(2.2)	<0.0001
Yes	1365	1273	(93.3)	92	(6.7)	
**Chronic disease**						
No	17,420	17,179	(98.6)	241	(1.4)	<0.0001
Yes	8442	8044	(95.3)	398	(4.7)	
**Depression**						
No	23,169	22,813	(98.5)	356	(1.5)	<0.0001
Yes	2693	2410	(89.5)	283	(10.5)	
**Year**						
2013	5433	5246	(96.6)	187	(3.4)	
2014	5362	5182	(96.6)	180	(3.4)	
2015	5230	5119	(97.9)	111	(2.1)	
2016	4974	4879	(98.1)	95	(1.9)	
2017	4863	4797	(98.6)	66	(1.4)	
Total	25,862	25,223	(97.5)	639	(2.5)	

* Separated marital status included divorced, separately living, and widowed individuals. Depression categorized based on the Center for Epidemiological Studies Depression (CES-D) 11 scale.

**Table 2:** Employment and other factors associated with suicide ideation.

	OR	95% CI
Employment transition *		
Permanent → permanent	1.00	
Permanent → precarious	1.74	(1.29–2.35)
Permanent → unemployment	1.97	(1.32–2.96)
Precarious → permanent	1.57	(0.73–3.36)
Precarious → precarious	1.86	(1.21–2.85)
Precarious → unemployment	1.43	(1.05–1.95)
Unemployment → permanent	1.68	(0.99–2.84)
Unemployment → precarious	1.11	(0.49–2.50)
Unemployment → unemployment	1.57	(0.86–2.87)
Employment status *		
Permanent	1.00	
Precarious	1.52	(1.17–1.99)
Unemployed	1.36	(1.05–1.78)
**Sex**		
Male	1.00	
Female	1.13	(0.92–1.40)
**Age**		
25–34	1.00	
35–44	1.10	(0.80–1.51)
45–59	1.13	(0.81–1.58)
**Region**		
Metropolitan cities	1.00	
Urban	0.98	(0.80–1.18)
Rural	0.69	(0.50–0.95)
**Education level**		
High school or below	1.00	
University or above	0.73	(0.58–0.92)
**Marital status**		
Single	1.00	
Separated **	1.08	(0.77–1.49)
Married	0.62	(0.47–0.81)
**Income**		
Low	1.00	
Medium	0.62	(0.50–0.79)
High	0.48	(0.36–0.63)
**Disability**		
No	1.00	
Yes	1.23	(0.90–1.68)
**Chronic disease**		
No	1.00	
Yes	2.08	(1.71–2.52)
**Depression**		
No	1.00	
Yes	3.19	(2.64–3.87)
**Year**		
2013	1.00	
2014	0.95	(0.76–1.19)
2015	0.58	(0.45–0.75)
2016	0.57	(0.44–0.75)
2017	0.42	(0.31–0.56)

* Employment transition and employment status were analyzed as two different models but with the same covariates as above. ** Separated marital status included divorced, separately living, and widowed individuals. Depression was categorized based on the CES-D 11 scale.
